# Urgent Eye Care in the UK Increased Demand and Challenges for the Future

**Published:** 2014

**Authors:** Thomas Siempis

**Affiliations:** Great Western Hospital, UK

## INTRODUCTION

Over the recent years, urgent eye care services in the UK have seen a big increase in the number of patients attending them. Literature confirms that eye casualty services routinely run in excess of their intended capacity (1-5). As a result, hospitals are struggling to keep up with the demand (6). The same is also true in other countries such as New Zealand (7). However, most of the “urgent eye” attendances are, actually, non-urgent problems, which could be potentially dealt in the community (5, 8-12). The high number of patients presenting to these services poses great challenges, logistical and clinical, for their efficiency (13). Hence, there is room for improvement. This can be achieved by carefully shifting these cases to the community and thereby ensuring a sustainable system of urgent eye care in the future. There is a fine balance between reducing inappropriate referrals and deterring primary healthcare professional from referring truly urgent cases.

The purpose of this review is to provide an overview of the urgent eye care in the UK and the problems it faces, based on published original research articles, audits and publications/guidances from the Royal Colleges of Ophthalmologists and the College of Optometrists. In the following sections, the structure of urgent eye care in the UK, the appropriateness and patterns of referrals along with the reasons for increased demand will be presented. Finally some proposals and established solutions will be described.

## STRUCTURE OF URGENT EYE CARE IN THE UK

Dedicated eye casualty departments, rapid access outpatient clinics and general Accident and Emergency (A&E) departments usually provide emergency eye service in the UK. GP and optometrists may also deal with urgent ophthalmic conditions (1). Some eye units, especially dedicated eye casualty departments, can offer direct access through a walk-in service (14). Most of these departments operate during normal working hours and outside these hours the patients need to visit the A&E (15). Studies have shown that reducing the ophthalmic casualty opening times to 9am till 5pm every day of the week, with after-hours cover provided by the on-call doctors at acute hospital A&Es, is sufficient (16). Depending on the needs of the local community, population and resources of the local primary care services, there are differences in the way the aforementioned services function (14). In London, Moorfields and the Western Eye Hospital are the only centers providing full 24 hours a day, 7 days a week cover of the walk in service. There are further six units, which provide an out-of-hours on-call ophthalmic service, for eye casualties attending through general A&E (1). Referrals can come from GPs, A&Es, optometrists and other hospitals, or patients can “self-refer” and present to these services (13). On arrival, patients are usually triaged by a nurse practitioner before seeing the ophthalmologists, if deemed necessary (13, 16).

## DISTINGUISHING URGENT FROM SIGHT THREATENING EYE CONDITIONS

The College of Optometrists and The Royal College of Ophthalmologists (RCOphth) define urgent eye condition as “any condition that is of recent onset and is distressing or is believed by the patient, carer or referring health professional to present an imminent threat to vision or to the general health” (6). On the contrary, an eye emergency is described as a condition that “its occurrence is unpredictable and it requires treatment or admission at short notice to avoid damage to the eye or eyesight” by the RCOphth (17). For this reason, a distinction should be made between urgent cases and true emergencies. Only a small proportion of acute eye conditions are sight threatening and need to be seen urgently by an ophthalmologist. Among others, these include mechanical injury to the globe, chemical injuries, central retinal artery occlusion, acute angle closure glaucoma and retinal detachment (18). The majority of other referrals could be appropriately referred to routine ophthalmology outpatient clinics or managed by GPs (3-5, 10).

 As a result, some of the eye conditions that are referred as urgent (with some being acute and others non-acute) are in reality non-sight or life threatening and can be managed by specialist trained GPs or optometrists. Some examples are blepharitis (non-acute), conjunctivitis, chalazion (non acute), dry eye (non-acute), contact lenses problems (non acute) and cataracts (non acute) (14).

On average 50-70% of referrals to these services are, actually, non-emergency cases (from now on referred as non-urgent cases) (14). Surprisingly, in some cases, referrals eventually classified as non-urgent make up to 78.1% of the total referrals (5). To be specific, an audit of primary care referrals in the ophthalmic A&E of the Royal Victoria Eye and Ear Hospital in Dublin in 2009 showed that those requiring urgent attention (should be seen in less than 60 minutes) and extremely urgent (should be seen in less than 10 minutes) made up 16.7% and 0.6% of the attendees, respectively. In other words, almost 80% of the patients did not require urgent consultation following triage (16). Fenton et al in 2001 found similar results when they studied the admissions in the ophthalmic division of an accident and emergency department in the UK. Again, non-urgent referrals constituted 60-70% of patients. Analysis of 265 referral letters from general practitioners to the A&E department of a large regional ophthalmology center in the UK showed that 50-70% of referrals did not constitute accidents or urgent conditions and could have been more properly assessed within usual secondary referral outpatient clinics (19).

## OUTCOME OF URGENT EYE CONSULTATIONS

Regarding the outcome of these consultations, most of the patients are discharged on the first visit and this is usually after being seen by the nurse practitioner alone. First visit discharges vary from 57%-62% (13, 14, 16). Referrals to sub-specialty were done on average in 12-36% of the cases (7, 10, 13, 14, 20). It is only a very small proportion of patients with urgent eye conditions requires admission and is usually around 1% (3). These results prove the point that urgent eye services are routinely used for non-urgent cases.

## PATTERN OF REFERRALS AND MOST FREQUENT DIAGNOSES

As most services operate a walk in service, most of the patients attending those services are self-referrals. GP referrals constitute the second most frequent source of referrals. See the table below for details. The reasons for the high percentage of self-referrals are discussed later in this review.

**Table 1.** Origin of referrals

	**A Hammed Al-Arrayedh et al. 2010 (13)**	**Wasfi et al. 2009 (21)**	**Hau et al. 2008 (14)**	**Fenton et al. 2001 (3)**
**Self referral**	-	34.2%	76.9%	54%
**GP referral**	16.6%	21.1%	8.1%	17.1%
**Optometrist referral**	-	-	10%	3.1%
**A&E referrals**	-	14.1%	-	-

In terms of the distribution of diagnostic groups, trauma (24%-65,6%), infection (26%-45.5%) and inflammation (12%-32.3%) are the most common ones (6, 16). Depending on the study, there is a variation in the proportional frequencies of the most common diagnoses. The most common diagnosis is conjunctivitis (10.4%) followed by foreign body, cyst, dry eye, uveitis, cataract, blepharitis, corneal abrasion, corneal ulcers and chemical injuries as well as blunt trauma (13).

## REASONS FOR INCREASED DEMAND

Emergency eye services are facing increased demand (6). This is confirmed by a recent study by HB Smith et al in 2013, where the effects of changes in the provision of emergency eye care in Central London were examined. According to this paper, patients’ volume in the ophthalmic A&Es at Moorfields Eye Hospital and Western Eye Hospital has risen particularly fast over the last 5 years, averaging 7.9% and 9.6% per year respectively (1). The authors of this paper attribute this increase to 3 parameters; a) the consolidation of out of ours service into fewer but larger units, b) the generalized increase in unplanned hospital attendances and c) London’s growing population.

The consolidation of out of ours service into fewer but larger units, is a result of the implementation of the European Working Time Directive (EWTD). EWTD states that the working week should be less than 48h by 2009, including doctors in training. This is a legal requirement. P. Black projected that 50% of UK eye departments had to abolish after hours work by 2009, in order to be compliant with EWTD and in line with the recommendations by the RCOphth (22, 23).

With respect to Moorfields emergency ophthalmic unit, another reason explaining the increased demand is that it operates a policy of never refusing a patient on the grounds of capacity. This guaranteed access is another factor, which might have led to an increased number of referrals over the past years (1).

The most important reason for increased demand of these services are the inappropriate referrals. Inappropriate referrals can be categorized in inappropriate self-referrals, GP referrals and A&E referrals:

*Self-referrals*

As mentioned above, self-referrals constitute the majority of the workload in eye casualty services. In general, people have a tendency of skipping their GP and visit A&E departments directly. The main reasons for that are the patients' perceptions of their problems and limited access to their GPs (24). The same is true in other countries such as the Netherlands, where 60% of the ED patients were self-referrals bypassing their GP for the same reasons (25). Other reasons include reduced waiting times in A&E (after the implementation of the 4 hour target) (26) and that the problem would be better managed by a specialist in the field. This is particularly true for specialist services, including eye services (27). 

There is an interesting article by Hau et al. published in 2008, which evaluated the reasons for attending a dedicated ophthalmic A&E department from the patients’ perspective (14). According to this study a variety of reasons makes a walk in casualty eye service or ophthalmic A&Es attractive. The most important one is the stress and anxiety created by the fear of visual loss. Sight is valued and sight loss is often feared. Studies have shown that eye diseases, which can cause blindness, may produce significant emotional distress and profoundly reduced quality of life (28). Furthermore, some eye conditions such as conjunctivitis and uveitis have a disfiguring nature, which increases anxiety too. What is more, patients are unable to identify which conditions are serious by their signs and symptoms and therefore feel the need to seek specialist advice urgently thereby bypassing their GP or optometrist (29). Finally immediate treatment and reassurance are rated more highly than diagnosis as the most important aspect of their urgent eye care (30).

*GP Referrals*

There is no recent literature regarding the appropriateness of GP referrals to emergency eye services in the UK. A study by Kheterpal in 1995 revealed that over 50% of GP referrals to an ophthalmic A&E department did not constitute true emergencies (18). In general, some healthcare professionals including GPs have a low threshold for referring patients to an ophthalmologist. This is due to lack of the relative equipment in General Practice such as slit lamps along with insufficient clinical skills or ophthalmic knowledge (31). Thus, compared with other specialties, a lot of primary care in ophthalmology takes place in hospitals rather than in general practice (32-35). There is a recent audit of primary care referrals to the Ophthalmic A&E department in the Royal Victoria Eye and Ear Hospital, in Dublin, Ireland by Al-Arrayedh et al. which showed that 80.9% of the referred patients by GPs were non-urgent cases. Non-painful and non-sight threatening conditions made up 51.3% of the total referrals. Furthermore, GP letters were found to have poor consistency of ocular examination. Only 16.4% had the visual acuity recorded and 51.1% did not record any examination findings (13).

*A&E Referrals*

As shown above, A&E referrals to casualty eye services constitute only a small part of the total referrals. It is obvious that the appropriateness of the referrals depends on the relative equipment of the A&E units, the training of their doctors in ophthalmic examination and their confidence in managing urgent eye conditions. There is an interesting study by Sim et al, published in 2007 in which 168 A&E departments were surveyed with respect to any changes in the management of eye emergencies across the UK between 1993 and 2003. It was found that the number of A&E departments with a slit lamp increased by 25.7%, A&E departments which provided slit lamp training increased by 21% and Senior House Officers (SHOs) who felt confident in using the slit lamp increased by 11.5%. Nevertheless, the proportion of SHOs who felt confident in dealing with these cases was not significantly different (31.2% in 1993 vs 36.1% in 2003, p = 0.36) (36).

**Table 2:** Reasons for Increased Urgent Eye Care Attendances/Referrals

**Consolidation of out of ours service into fewer but larger units due to ETWD.** **Never refusing a patient policy by some units.** **Inappropriate self-referrals (mainly self referrals due to stress and anxiety by the fear of loss of vision).** **Low threshold for referrals by GPs or A&E departments due to lack of equipment for complete ophthalmology examination and/or low confidence in managing eye conditions due to lack of training and thus confidence**




A&E: Accident and Emergency; SHO: Senior House Officers; ETWD: European Time Working Directive; GPs: General Practitioners.

## PROPOSALS TO MAINTAIN EFFECTIVE AND SUSTAINABLE URGENT EYE CARE

Up to now various studies have addressed the problem of increased demand in urgent eye care services. In these studies, several proposals have been made that aim to make urgent eye care more sustainable in the UK. These include the recruitment of ophthalmic nurse practitioners in casualty eye services/ ophthalmic A&Es, the use of optometrists as first point of contact in urgent cases either in the hospital or in the community, the enhancement of the GP training in ophthalmology and many more. The future lies in the shift of care to the community in order to use NHS resources more effectively. These should be combined with regular clinical audits of the casualty eye service and the introduction of clinical management protocols in order to achieve a high standard of care.

## TO BE SPECIFIC

*Nurse Practitioners*

Various studies have shown that nurses are able to manage safe and effectively eye casualties (12, 37-39). Their contribution is invaluable in reducing the workload of ophthalmic and general A&E departments (12, 40, 41). A study conducted in the A&E department of St Bartholomew’s and The Royal London NHS Trust has shown that Emergency Nurse Practitioners (ENPs) were more accurate than A&E SHOs in obtaining history, measuring visual acuity and making provisional diagnoses (42). It has also been suggested that nurses with specialist training who follow concise protocols can offer an important service in triaging, assessing and treating common eye casualty presentations (37). 

Moorfields Eye Hospital has trained 25 Emergency Nurse Practitioners who are seeing 17% of casualty attendances overall. More specifically, all patients attending Moorfields A&E are triaged by nurses followed by an initial visual assessment. They are then referred to the ENP pathway where a trained nurse examines, treats and discharges a patient following a protocol. If any doubt arises, the patient is then referred to the doctor. In addition, Moorfields uses a scheme known as ‘Patient Group Directive’ (PGD). This allows limited prescribing powers to nurses who are not independent prescribers for the treatment of specified groups of patients with specific diagnoses according to a protocol casualty as well as the prescribing of chloramphenicol 1% ointment, chloramphenicol 0.5% drops, and hypromellose 1% drops when deemed appropriate (1).

*Better Training of GPs in Ophthalmology*

GPs will manage most of the eye problems that are presented to them, despite having uncertainties (6). Nevertheless, a significant proportion of the referrals to casualty eye services comes from GPs as describe above. Since the majority of ophthalmic cases presented to GPs needs either external examination of the eye or of the anterior segment, this emphasizes the need for the acquisition of these skills both in the undergraduate and postgraduate level (35). Training in the use of slit lamp and in management of common urgent eye conditions such as the removal of corneal foreign bodies, chalazion, red eye or chronic lid infection will reduce the misuse of the casualty eye service (21). For this to happen, extra funding should be given in order to ensure that GPs along with optometrists gain the necessary training (14) by attending various clinics such as eye casualty, medical ophthalmology, neuro-ophthalmology and external eye disease clinics (31). Finally, ophthalmology study days could enhance their continuing education in ophthalmology (13). The aforementioned will help GPs avoid diagnostic uncertainties, which could cause inappropriate referrals and increase their confidence in managing common ophthalmological problems.

Another useful proposal is the utilization of a standard referral form to the casualty eye services, which will contain questions such as the presenting complaint, as well as, findings from simple objective examinations such as the measurement of visual acuity and external eye examination. These are critical for an effective nursing triage and eventually quicker assessment of the patient (13).

Last but not least, the introduction of a registry of GPs with a special interest in ophthalmology as suggested by the Royal College of Ophthalmologists is another helpful idea which shifts the service to the community and reduces cost. These GPs should have a minimum qualification such as DRCOpth and follow some recognized standards for Continuous Professional development as well as revalidation (31).

This proposal will only be successful if the patients are educated, encouraged to look at this list and consult their local practitioner with an interest in ophthalmology before being referred by other GPs or self- referred to the hospitals (14).

*Optometrists*

“Optometrists are primary health care specialists trained to examine the eyes and detect defects in vision, signs of injury, ocular diseases or abnormality and problems with general health” as defined by the College of Optometrists (43). Sometimes community optometrists are wrongly considered as shops for spectacles rather than an eye health check (31). Nevertheless, optometrists provide more primary eye care than GPs especially by managing refractive errors as well as screening for glaucoma. They have been characterized as the gatekeepers of most referrals to the hospital eye service (HES) via the GP and for some conditions directly to HES (44, 45). In a number of hospitals, the shared care of patients by optometrists and ophthalmologists is well established (46-49). Optometrists can be supplementary prescribers or independent prescribers or can also operate under a PGD (6).

Hau et al. did a prospective 6 month study at Moorfields eye hospital which assessed the percentage agreement for primary, secondary diagnosis and management between senior optometrists and consultants ophthalmologists. The results demonstrated good agreement both for primary diagnosis (89.3%) and referral management (90.7%). The consultant ophthalmologists found that 45.5% of the cases could have been seen only by an optometrist (50). 

## EXAMPLES OF SUCCESSFUL ESTABLISHED SCHEMES

In Wales, an innovative scheme called PEARS (Primary Eyecare Acute Referral Scheme) was introduced in 2003 (6). Patients who require a PEARS examination, either self-refer or are being referred by their GP and they are seen by optometrists within 24 hours of the request. Optometrists who participate in this scheme have passed theoretical and practical assessments to improve their skills in recognizing and managing ocular disease. They perform the necessary and appropriate examinations in order to reach a diagnosis. This service is free of charge to the patient and optometrists, who are the sole providers of this service, receive a fee of 38 pounds (51). Three years after the introduction of this service a relative study was published in the British Journal of Ophthalmology. It evaluated the efficacy of this scheme and showed that (66%) of all individuals were managed in optometric practice without being referred to their GP or Hospital Eye Services (HES). Only 1% of the individuals were found to have been inappropriately managed. 75% of the referrals to the HES were appropriate (51).

An example of the combination of the above is the formation of the Grampian Eye Health Network. Following the dramatic findings of an audit of the walk in service at Aberdeen’s eye department, where over 90% of the cases could have been treated by someone other than a doctor, several changes were implemented to improve the service (6). These included the introduction of a 24h eye health network line, the establishment of afternoon consultant led eye assessment clinics and the extension of the Patient Groups Directive (52).

## CONCLUSIONS

Without doubt, the increased strain on urgent eye care service affects other parts of eye clinics too, as it deprives them of valuable human and financial resources. As described above there are several reasons for that with inappropriate referrals being the most important one. This capacity problem should be an opportunity to increase efficiency in casualty eye services as several schemes have successfully achieved. Examples include the PEARS system in Wales, the example of NHS Grampian in Scotland and the various changes in the function of Moorfields ophthalmic A&E. On balance, regular audits, the increased use of ophthalmic nurse practitioners, optometrists and better training of GPs, whose common characteristic is the shift of eye care to the community, will provide a more sustainable future for urgent eye care services.
